# A novel *HADHA* variant associated with an atypical moderate and late-onset LCHAD deficiency

**DOI:** 10.1016/j.ymgmr.2022.100860

**Published:** 2022-03-15

**Authors:** Anne-Frédérique Dessein, Eléonore Hebbar, Joseph Vamecq, Elodie Lebredonchel, Aurore Devos, Jamal Ghoumid, Karine Mention, Dries Dobbelaere, Marie Joncquel Chevalier-Curt, Monique Fontaine, Sabine Defoort, Vassily Smirnov, Claire Douillard, Claire-Marie Dhaenens

**Affiliations:** a Univ. Lille, CHU Lille, Centre de Biologie Pathologie Génétique, UF Métabolisme Général et Maladies Rares, F-59000 Lille, France; b CHU Lille, Cardiology Department, F-59000 Lille, France; c Inserm, Biochemistry and Molecular Biology Laboratory, HMNO, CBP, CHRU Lille & EA 7364 – RADEME, North France University Lille, F-59000 Lille, France; d Univ. Lille, CNRS, UMR 8576 – UGSF - Unité de Glycobiologie Structurale et Fonctionnelle, F-59000 Lille, France; e Univ. Lille, CHU Lille, Pôle Biologie Pathologie Génétique, Institut de biochimie et de biologie moléculaire, UAM de Glycopathologies, F-59000 Lille, France; f CHU Lille, Centre de Biologie Pathologie Génétique, UF Génopathies, F-59000 Lille, France; g CHU Lille, Clinical Genetics Department, Reference Center for Developmental Anomalies, F-59000 Lille, France; h Medical Reference Center for Inherited Metabolic Diseases, Jeanne de Flandre University Hospital and RADEME Research Team for Rare Metabolic and Developmental Diseases, EA 7364 CHU Lille, F-59037 Lille, France; i CHU Lille, Exploration of Vision and Neuro-ophthalmology department, Lille University Hospital, F-59000 Lille, France; j CHU Lille, Department of Endocrinology and Metabolism, F-59000 Lille, France; k Univ. Lille, Inserm, CHU Lille, U1172-LilNCog-Lille Neuroscience & Cognition, F-59000 Lille, France

**Keywords:** LCHAD, Late-onset, Atypical maculopathy, Cardiomyopathy, HADHA, Mitochondrial trifunctional protein MTP

## Abstract

**Background:**

Long chain 3-hydroxyacyl-CoA dehydrogenase deficiency (LCHADD) is a rare inherited disease caused by pathogenic variants of *HADHA* gene. Along with signs common to fatty acid oxidation defects (FAOD), specific retina and heart alterations are observed. Because long-chain fatty acid oxidation is selectively affected, supplementations with short/medium-chain fats represent energetic sources bypassing the enzymatic blockade. Here, we report on an atypical presentation of the disease.

**Methods:**

Clinical features were described with medical explorations including ophthalmic and cardiac examination. Biological underlying defects were investigated by measurements of biochemical metabolites and by fluxomic studies of mitochondrial β-oxidation. Whole exome sequencing and molecular validation of variants confirmed the diagnosis.

**Results:**

The patient has developed at nine years an unlabeled maculopathy, and at 28 years, an acute cardiac decompensation without any premise. Blood individual acylcarnitine analysis showed a rise in hydroxylated long-chain fatty acids and fluxomic studies validated enzyme blockade consistent with LCHADD. Genetic analysis revealed the common p.(Glu510Gln) variant in *HADHA*, in *trans* with a novel variant c.1108G > A, p.(Gly370Arg) located in the NAD binding domain. Patient pathology was responsive to triheptanoin supplementation.

**Conclusion:**

This atypical LCHADD form report should encourage the early assessment of biochemical and genetic testing as a specific management is recommended (combination with fast avoidance, low fat-high carbohydrate diet, medium-even-chain triglycerides or triheptanoin supplementation).

## Highlights

• Mild hyperpigmented macular dots could be the first and early symptom of moderate LCHAD.
• The novel *HADHA* c.1108G > A, p.(Gly370Arg) is hypomorphic and associated with moderate LCHAD.
• Atypical and late LCHAD can be deciphered by joint biochemical and genetical investigations.
• Acylcarnitines must be tested in unexplained macular dystrophy and/or dilated cardiomyopathy.
• Supplementation with the triglyceride triheptanoin is effective.

## 1 Introduction

Long-chain 3-hydroxyacyl-CoA dehydrogenase (LCHAD) deficiency (OMIM *609016) is a rare metabolic disease among all fatty acid oxidation disorders. LCHAD activity is one of the three enzymatic activities of mitochondrial trifunctional protein (MTP), a protein complex anchored in the mitochondrial inner membrane. MTP catalyzes the last three steps of mitochondrial long-chain fatty oxidation, including enoyl-CoA hydratase, 3-hydroxyacyl-CoA dehydrogenase, and 3-ketoacyl-CoA thiolase activities. MTP is a hetero-octamer consisting of four alpha and four beta subunits, respectively encoded by the *HADHA* (chr2, NM_000182.4) and *HADHB* (chr2, NM_000183.2) genes. The alpha subunit harbors both enoyl-CoA hydratase and 3-hydroxyacyl-CoA dehydrogenase activities, and the beta subunit harbors 3-ketoacyl-CoA thiolase activity [1], [2].

Two distinct metabolic disorders have been described: LCHAD and MTP deficiency. Of the two, LCHAD deficiency is the most frequent. LCHAD deficiency refers to the loss of 3-hydroxyacyl-CoA dehydrogenase activity alone, whereas mitochondrial trifunctional protein deficiency refers to the loss of 3-hydroxyacyl-CoA dehydrogenase activity and the other 2 long-chain acyl-CoA β-oxidation enzymes activities. However, metabolic screening investigations cannot distinguish these forms, and diagnoses can only be confirmed by gene sequencing. The most common gene variant causing LCHAD deficiency is c.1528G > C p.(Glu510Gln) in the *HADHA* gene [1], [2], [3].

Isolated deficiency of long-chain 3-hydroxyl-CoA dehydrogenase is an autosomal recessive disorder presenting with hypoglycemia, hepatopathy, cardiomyopathy, muscle hypotonia, neuropathy, retinopathy and fat accumulation in affected tissues. Typical presentations vary according to age of onset and severity: a very early-onset neonatal and severe form with rapid worsening, an infantile mainly hepatic form, and a milder late form associating neuropathy and myopathy. Symptoms are usually worsened by prolonged fasting and/or insufficient glucose relative to increased demand during acute illness [3], [4]. Among clinical signs, hypoketotic hypoglycemia is a classical feature of all fatty acid oxidation disorders (FAOD), liver dysfunction and cardiomyopathy are indicative of long-chain FAOD and retinal disease is pathognomonic of LCHAD/MTP deficiency. Ophthalmological findings in patients with LCHAD range from normal or pale fundus to total pseudo-colobomatous chorio-retinal atrophy [5]. Macular depigmentation, clumping and loss of retinal pigment epithelium are early changes in the pathogenesis of LCHAD retinopathy. LCHAD patients are usually diagnosed at a later stage characterized by pigment aggregates with or without areas of surrounding hypopigmentation at the level of the retinal pigment epithelium (RPE), especially in the macula and more rarely in midperipheral retina [6], [7]. Fatty acid oxidation (FAO) enzymes are highly expressed in the RPE and other ocular structures (extraocular and intraocular muscle, corneal endothelium, and ciliary epithelium) and active fatty acid oxidation was proven in a cultured porcine RPE cell model [8]. This pathway plays a key role in ocular energy production *in vivo* [8], [9]. Recently, in a hiPSC-derived RPE cell model, Polinati et al. showed that accumulation of long-chain fatty acid derivatives can impair normal cell signaling, induce peroxidation, and cause cellular dysfunction and apoptotic cell death [7]. At the RPE level, deformed cell-to-cell adhesion and abnormal tight junctions weakened the blood-retina barrier possible contributing to the specific degeneration of the RPE layer observed in LCHAD deficiency [7].

In the present study, we report an atypical presentation of LCHAD in a patient initially presenting with maculopathy during childhood and LCHAD diagnosis only established in the patient's thirties upon the evolution towards severe dilated cardiomyopathy and retinopathy.

## 2 Materials and methods

### 2.1 Ophthalmic clinical evaluation

The patient was initially followed at the Lille University Hospital Neuro-Ophthalmology and Visual Explorations unit. He underwent ophthalmologic examination including visual acuity (VA) testing, Goldmann visual field testing, refraction, slit-lamp examination, dilated fundus examination, fundus photography, fundus autofluorescence (FAF), optical coherence tomography (OCT), full-field flash electroretinography (ERG) and electrooculogram (EOG) recorded according to the guidelines of the International Society for Clinical Electrophysiology of Vision [10].

### 2.2 Biochemical analysis and functional assessment

The patient was subsequently followed in the Cardiology and Endocrinology-Metabolism units of the Lille's University Hospital. Urinary organic acid profiling and blood acylcarnitine profiling are part of routine metabolic screening performed daily in metabolic units. Fluxomic study of the *de novo* synthesis of acylcarnitines in whole blood samples incubated with deuterated palmitate was performed as described previously [11].

### 2.3 Whole-exome sequencing and molecular validation of the variants

Genomic DNA of the patient and his parents was isolated from 5 ml peripheral blood using the DNA Blood 1 k Kit on the automated workstation Chemagic Star (PerkinElmer, Waltham, MA, USA). The molecular diagnosis adhered to the tenets of the Declaration of Helsinki. Written informed consent was obtained from the patient and his parents for the segregation study.

The mitochondrial genome was amplified according to the Revised Cambridge Reference Sequence (rCRS) of the Human Mitochondrial DNA NC_012920.1. Two separate fragments were amplified (first fragment from nucleotide m.9850 to m.9339; second fragment from m.15698 to m.14861) using the TaKaRa LA Taq kit (Takara Clontech, Mountain View CA, USA). DNA libraries were prepared using the Illumina Nextera XT DNA library preparation kit (Illumina, San Diego, CA) according to the manufacturer's protocol and sequenced on a MiSeq (Illumina, San Diego, CA, USA). The generated sequences were analyzed using Seqnext (JSI Medical System, Ettenheim, Germany) and MITOMAP servers.

Exome sequencing was performed by IntegraGen SA (Evry, France) using the Sureselect XT clinical Research Exome kit (Agilent Technologies, Santa Clara, CA, USA) with the biotinylated oligonucleotides probes library (SureSelect XT Clinical Research Exome - 54 Mb, Agilent), followed by paired-end 75 bases massively parallel sequencing on Illumina HiSeq4000 (Illumina, San Diego, CA, USA). Image analysis and base calling were performed using Illumina Real Time Analysis (2.7.3) with default parameters. Sequence reads were mapped to the human genome build (hg19 / GRCh37) using Elandv2e (Illumina, CASAVA1.8.2). Variants filtering was done with the in-house pipeline ERIS V3 tool (IntegraGen). The mean depth for the proband analysis was 107×, 87% of sequences had a 25× coverage and 97.38% of sequenced bases had a quality score greater than or equal to Q30. Candidate variants were filtered based on function and minor allele frequency (< 1%).

To confirm the presence of the variant, we Sanger sequenced the exons 12 and 15 of *HADHA* gene (OMIM *600890, NM_000182.4). Fragments were amplified under standard polymerase chain reaction (PCR) conditions. PCR products were purified on P10 gel (Bio-Rad, Hercules, CA, USA) and sequenced in sense and antisense directions using the BigDye® Terminator v3.1 Cycle Sequencing Kit on a 3730 DNA analyzer (Applied Biosystems, Carlsbad, CA). Primer sequences and PCR conditions are available upon request. To evaluate the pathogenicity of the identified missense variants, we used several programs: Polymorphism Phenotyping v2 (PolyPhen-2) (http://genetics.bwh.harvard.edu/pph2/); Sorting Intolerant From Tolerant (SIFT) (http://sift.jcvi.org/) and a-GVGD through Alamut Prediction software and many others through VarSome (The Human Genomic Variant Search Engine) (https://varsome.com). A total of twelve softwares have been assessed: Polyphen, SIFT, a-GVGD, Condel, DANN, GERP, FATHM, LRT, MetalLR, MetaSVM, MutationAssessor and Provean.

## 3 Results

### 3.1 Case description

A young boy presented with photosensitivity starting at the age of four, with discreet erythematous and pruritic edema of the face and back of the hands. Neither bullous nor scarring lesions were observed. Given the presentation, erythropoietic protoporphyria was initially suspected and he was treated with canthaxanthine despite normal globular protoporphyrin dosage. At age nine, he was referred to the Lille University Hospital Ophthalmology clinic to monitor the ocular toxicity of this treatment. Fundus examination revealed subtle hyperpigmented macular dots and a normal peripheral retina. Despite these visible macular changes, best-corrected visual acuity and functional tests (static and kinetic visual field, full-field ERG and EOG) were normal. Such findings are unusual in both erythropoietic protoporphyria and canthaxanthine retinal toxicity. The cutaneous symptoms of presumed erythropoietic protoporphyria were very mild and canthaxanthine was discontinued after 1 year of treatment. Over 10 years of ophthalmologic follow-up, macular disease progressed very mildly. Visual discomfort of the patient appeared progressively by the patient's twenties and a slight visual acuity loss in his thirties. The patient's medical history was uneventful until the age of 25 apart from appendectomy and spontaneously resolving hepatitis (without further details and lacking liver biopsy).

At the age of 25, the patient presented intense myalgia after a period of intense physical work leading to hospitalization through the emergency department (ED). The patient worked as a workshop manager in an auto parts factory and had to carry heavy loads. Unfortunately, CPK were not assessed. Symptoms resolved with rest.

At the age of 28, a sudden worsening was noted, associating intense weakness, breathing difficulties and congestion. Echocardiography revealed chronic heart failure (CHF) due to dilated cardiomyopathy with low left ventricular ejection function (25%). Management of CHF was initiated with low doses of beta-blockers, angiotensin receptor blockers and mineralocorticoid receptor antagonists. In parallel, retinal changes became more characteristic with the appearance of a dark foveal dot and posterior pole depigmentation in radial and reticular pattern.

At the age of 30, the patient was hospitalized for cardiac re-evaluation. The patient was class II of the New York Heart Association's CHF classification (NHYA) and required diuretics. Cardiomyopathy with reduced left ventricular ejection function (35–40% according to cardiac ultrasound, 33% according to cardiac magnetic resonance imaging) with a restrictive profile. Functional capacity was reduced with a peak oxygen consumption (VO_2_) at 55% of the maximal predicted value. NT-proBNP (cardiac peptide amino-terminal brain natriuretic peptide) was high at 2025 pg/ml. Right heart catheterization (RHC) showed post-capillary hypertension worsening with dobutamine use. Following treatment optimization, the patient was selected for heart transplantation.

The context of potential heart transplant in a young patient lacking a clear etiology of CHF, led to clinical reevaluation. An in-detail retrospective review of the patient's neonatal and pediatric history did not reveal any features suggestive of a specific metabolic disorder. His parents and sister were healthy, and there was no family history of sudden infant death syndrome (SIDS). Nothing was reported during pregnancy and delivery: specifically, his mother presented neither preeclampsia, Hemolysis Elevated Liver enzymes Low Platelet (HELLP) syndrome, nor Acute Fatty Liver of Pregnancy (AFLP). His birth size was normal and neonatal history was devoid of hypoglycemia or metabolic disorders. Psycho-motor development was normal. The patient presented neither muscular nor neurological pain during catabolic conditions such as fasting or undercurrent infectious diseases. Until the age of 25, he had a healthy active lifestyle including high-intensity physical activity lasting more than an hour (running, trail running, and competitive tennis) without any reported muscle pain, cramps, cardiac event or other signs suggestive of myoglobinuria or rhabdomyolysis, such as dark urine.

### 3.2 Biochemical results and functional assessment

**Fig. 1:**
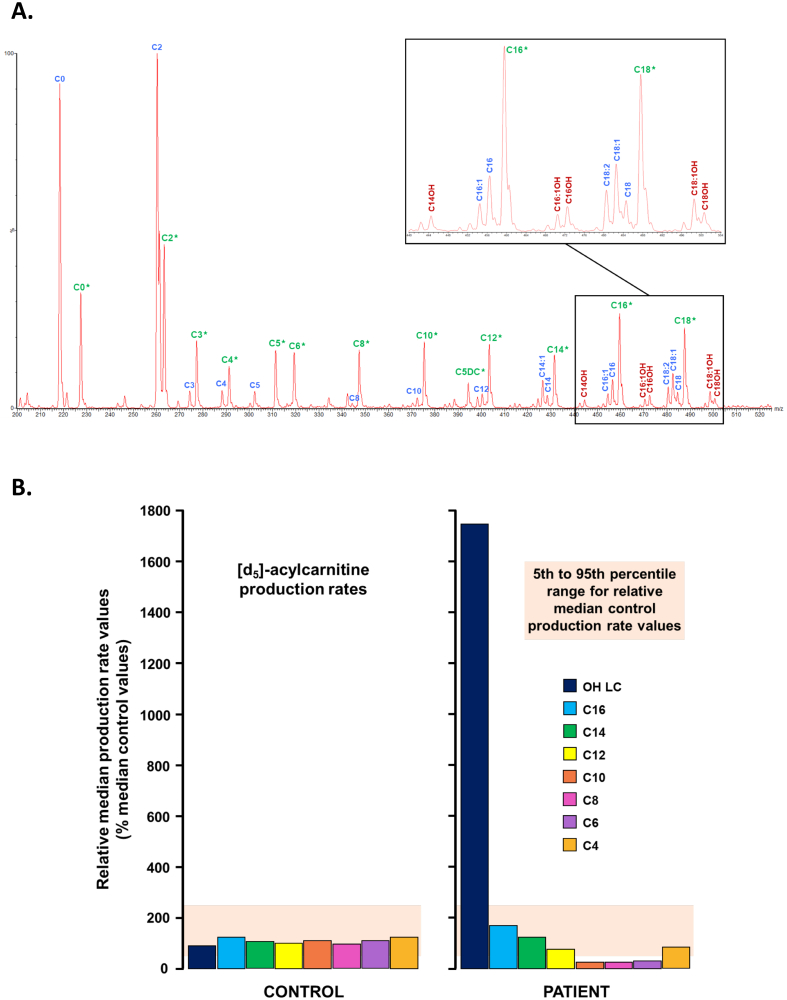
Individual acylcarnitine analyses in the patient. **A. Diagnostic serum acylcarnitine non-fluxomic profile.** Acylcarnitines were directly assayed in serum from the patient without any prior added substrate or incubation. Acylcarnitine from the patient are written in blue, and corresponding internal standards are indicated in green. Abnormal hydroxylated acylcarnitines are written in dark red. The presence and increase of hydroxylated forms of long-chain fatty acylcarnitines is evocative of LCHAD deficiency, and have prompted *ex vivo* fluxomic studies in order to explore more in-depth this diagnosis. **B. *Ex vivo* fluxomic study of the mitochondrial** β**-oxidation on whole blood.***De novo* individual acylcarnitine synthesis from deuterated palmitate incubated with whole blood samples from our patient *versus* a healthy control is illustrated.

After 16 h of fasting, neither hypoglycemia nor an increase in serum CPK were detected. Further biochemical metabolic screening aimed to assess carnitine status and fatty acid oxidation pathway. Quantitatively, urinary excretion of organic acids was normal as well as blood levels of total, free and esterified carnitine. In contrast, qualitative abnormalities in some individual carnitine esters were observed, consisting in a 2 to 3-fold increase in the hydroxylated forms of long-chain fatty acylcarnitine including 3-hydroxymyristoyl-, 3-hydroxypalmitoyl-, 3-hydroxypalmitoleyl-, 3-hydroxystearoyl- and 3-hydroxyoleyl carnitine esters. Blood levels of other carnitine esters were in the normal range. This profile led to suspect alterations in LCHAD activity of the mitochondrial trifunctional protein (MTP), despite an accumulation of long-chain 3-hydroxy-acylcarnitines lower than usually observed in typical early-onset phenotypes (Fig. 1A). This suspicion was further assessed by fluxomic measurements of *de novo* deuterated acylcarnitine synthesis in whole blood incubated with deuterated palmitate as a substrate (11−13). Results showed dynamic excess yield of deuterated 3-hydroxypalmitoyl-carnitine associated with a lack of downstream medium- and short-chain acylcarnitine production (Fig. 1B). This fluxomic pattern of acylcarnitine species production unequivocally confirmed a LCHAD/MTP deficiency.

### 3.3 Genetic assessment

The first genetic investigations were conducted at the age of 29, requested by the Ophthalmology department. As the fundal aspect associated with cardiomyopathy suggested mitochondriopathy, whole mitochondrial DNA was sequenced but no pathogenic variant was found. Whole exome sequencing (WES) was then performed. No variation was found in genes involved in maculopathies.

**Fig. 2:**
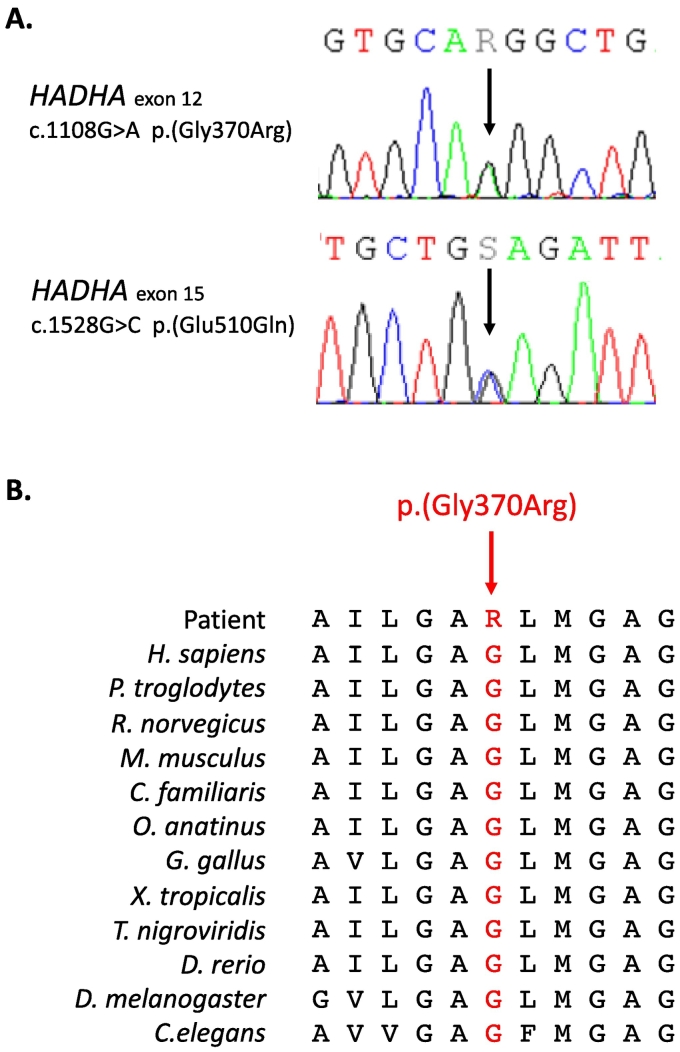
**Description of the two *HADHA* variants identified. A. Electropherograms of DNA sequencing.** The c.1108G > A p.(Gly370Arg) variant is located in exon 12 and corresponds to a G to A nucleotide substitution. **B. Cross-species protein conservation** flanking p.(Gly370Arg).

**Fig. 3:**
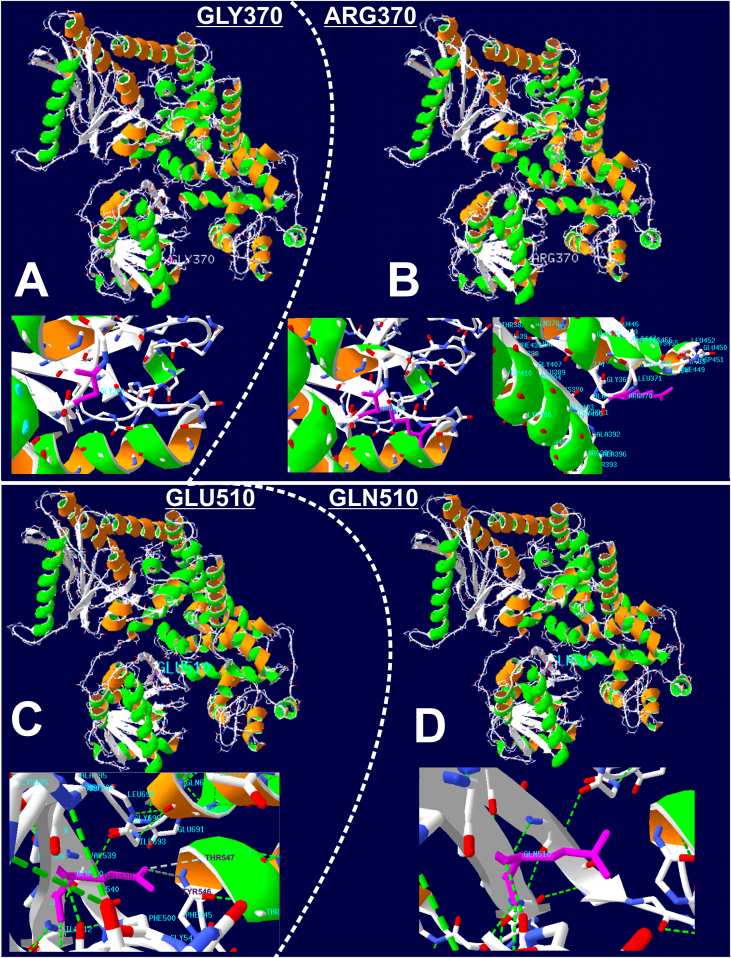
**3D modeling of the p.(Gly370Arg) and p.(Glu510Gln) variants and impact on HADHA chain A conformation**. The 3D structures of the novel variation p.(Gly370Arg) (A and B) and of the p.(Glu510Gln) (C and D) have been modelled with the swissmodel.expasy.org website (https://swissmodel.expasy.org) through upload of the FASTA sequences of wild and muted proteins. Panels A and B illustrate the occupancy at position 370 by glycine and arginine respectively (in purple). The replacement of a small uncharged residue (Gly370, A) by a bulky and charged one (Arg370, B) is expected to impact consequently protein structure and function. The extent of this impact might be partially attenuated but not cancelled by the displacement of the arginine at the periphery of the protein, thus minimizing steric hindrance with neighboring amino acid residues. The replacement of glutamate in position 510 (C) by glutamine (D), leads to the loss of hydrogen bonds (in dotted green lines) established between Glu510 and Thr547 or Tyr546. The location of this variant being internal, and not peripheral in contrast to p.(Gly370Arg), the loss of these hydrogen bonds should seriously impact protein stabilization and structure, and hence protein function.

In parallel, the biochemical fluxomic data was obtained in relation to reassessment of the patient's cardiomyopathy in the context of possible transplantation, indicating dysfunction of 3-hydroxyacyl-CoA dehydrogenase/3-ketoacyl-CoA thiolase/enoyl-CoA hydratase activities. This led to direct assessment of the *HADHA* gene on the same whole exome sequencing and two variants were found (Fig. 2). These variants were the recurrent well-known c.1528G > C, p.(Glu510Gln) variant located in exon 15 and an unknown new variant c.1108G > A, p.(Gly370Arg) located in exon 12. This variant occurs in a conserved sequence of an important functional domain of the protein, the NAD binding domain. Modeling of the protein variants is illustrated in Fig. 3. According to the *American Medical College of Genetics and Genomics (2015*) recommendations [12], the p.(Gly370Arg) was considered as a likely pathogenic variant (class 4). Indeed, it was not found in gnomAD exome despite a good coverage (x 91), twelve *in silico* analysis softwares used predicted it to be pathogenic. The familial study confirmed the bi-allelic segregation of the 2 variants, allowing the confirmation of the LCHAD diagnosis, linked to the *HADHA* gene. The involvement of these two variants in the disease of our patient was proven by abovementioned functional enzymatic testing.

Finally, WES results were used to search for the molecular basis of the erythropoietic protoporphyria previously suspected during childhood. However, no variant was found in *FECH, ALAS2* and *CLPX* genes to prove this disease.

### 3.4 Evolution under treatment

Specific metabolic management of LCHADD was initiated consisting in 1) restricting periods of fasting, 2) a diet limited in long-chain triglycerides (“low fat-high carbohydrate diet”) combined with supplementation in medium-even-chain triglycerides (MCT) 40 g per day. After 2.5 months, MCT supplementation was replaced by triheptanoin, a triglyceride of medium-chain fatty acids esterified with heptanoate, based on the published report of beneficial effects at time of diagnosis [13]*.* Triheptanoin was introduced gradually to avoid digestive intolerance: initially at 20 g/d for 15 days, then 40 g/d, and finally reaching 60 g/d a year after treatment initiation. The 60 g/d maintenance dose amounted to 26% of total daily caloric intake total (DCI). DCI was 2825 kcal/d (45 kcal/kg/d), including both 22 g/d natural fat intake (long-chain fatty acids, for essential fatty acid requirements) and 60 g/d triheptanoin. In this context of chronic heart failure, sodium beta-hydroxybutyrate at 2.5 g/qid (10 g/d) was subsequently introduced in association with a low salt diet (4 g/d) with very good tolerance.

This specific metabolic management was associated with a significant improvement of the acylcarnitine profile two weeks after initiating the changes in diet.

In parallel, cardiovascular treatment regimen was reinforced: introduction of valsartan with gradual increase in dosage to 160 mg/d, doubling of the dosage of furosemide to 80 mg/d, and maintenance of beta-blocker (2.5 mg/d) and eplerenone (25 mg/d) at the same dosages. Associated with the change in diet, this regimen resulted in improving cardiovascular status during follow up: decreasing symptoms of heart failure, decreasing NT-proBNP, and lower pulmonary pressures resulting in the patient being taken off the transplant waiting list. After one year of treatment, left ventricular ejection function was improved (50%).

## 4 Discussion

To our knowledge, we describe for the first time an atypical form of LCHAD initially presenting as mild progressive maculopathy since the age of 9 years and diagnosed upon rapidly progressing chronic heart failure as an adult. This rare metabolic condition was revealed during a workup when heart transplant was being considered for this patient, allowing diagnosis, specific management and rapid patient improvement avoiding heart transplant.

Clinical diagnosis of LCHAD was particularly difficult to establish at disease onset due to initial atypical retinal dystrophy and atypical subsequent clinical presentation. During childhood, neither the initial aspect of the retinal dystrophy nor its slow progression was typical of LCHAD. Usually, retinal changes of LCHAD are observed very early, during the first years of life and progress rapidly and ultimately lead to end-stage retinopathy with total loss of RPE (Stage IV) reached by adulthood [3], [14]. While the retinopathy was only diagnosed at 9 years after cutaneous signs evoking erythropoietic protoporphyria led to treatment with canthaxanthine which has retinal toxicity, this treatment was only briefly prescribed with incomplete observance and it is unlikely that it caused or had an effect on retinopathy progression. The clinical presentation associating slowly progressing visual symptoms starting during childhood and rapidly progressing chronic heart failure as an adult did not fit any of the typical presentations of LCHAD: rapidly worsening severe neonatal form, mainly hepatic infantile form, and milder neuromyopathic late form. Furthermore*,* the patient lacked signs evocative of LCHAD or other FAODs. Indeed, there were no episodes of hypoketotic hypoglycemia, hyperlactacidemia, nor rhabdomyolysis despite a very active lifestyle and regular high-intensity sports. Despite a history of hepatic cytolysis which can be related to LCHAD, hepatic biopsy was normal [2], [15]. Overall, the single irrefutably typical sign was severe cardiomyopathy.

Several hypotheses may have contributed to this atypical form of LCHAD. First, the moderate phenotype is consistent with metabolic profile. Indeed, the ratio between characteristically accumulating compounds and downstream metabolites was ten times higher than observed in controls, but three times lower than expected in early-onset cases (unpublished data).

**Fig. 4:**
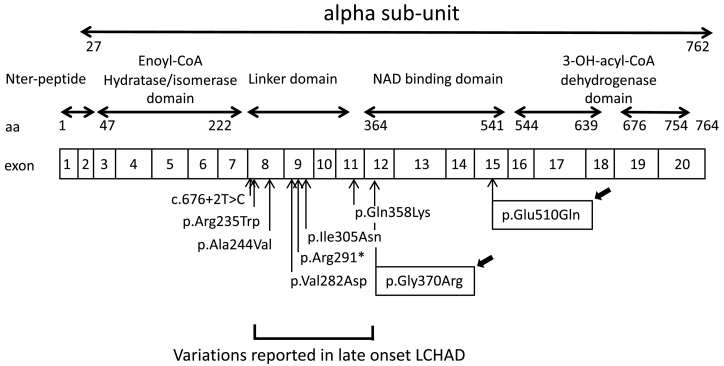
**Schematic representation of *HADHA* gene and localization of protein domains and late-onset associated variants.** Variations reported in late-onset LCHAD are located mostly in the linker domain encoded by exons 8 to 11. The novel p.(Gly370Arg) is located in close vicinity of this former domain, at the 5′ end of the exon 12 encoding the N-terminal end of the NAD binding domain. aa, amino acid; Nter, N-terminal.

Second, the moderate phenotype could also be consistent with the patient's genotype. He is compound heterozygous for two missense variants, one of which being the most frequent variation in *HADHA*: p.(Glu510Gln). This variant has already been described in classical phenotypes. It has been found both at the homozygous state (infantile hepatic forms) [1], [2], [15] and in compound heterozygous state. When *in trans* with a frameshift variant, it is associated with severe neonatal or hepatic forms, when *in trans* with a missense variant, it is associated with late-onset neuromyopathic form [1]. Previous descriptions of association with a later onset were all uncommon presentations of the disease. One was a 48-year-old patient who presented with recurrent rhabdomyolysis, heterozygous carrier for the c.1528G > C, p.(Glu510Gln) and the c.180 + 3A > G, p.(Thr37Serfs*6), leading to an exon 3 skipping and a premature stop codon [16]. A weak or moderate level of *HADHA* mRNA, due to a partial skipping of exon 3, could explain a mild expression of the disease. Other variants have been reported in late-onset (Fig. 4). Olpin et al. [17] described the p.(Ala244Val) *in trans* with a splice variation, in a patient with a mild form of episodic rhabdomyolysis from early childhood and peripheral neuropathy investigated at 29 years of age but initially examined at 3 years for a slowly progressive sensorimotor polyneuropathy with muscle weakness. An atypical presentation was also reported in a 27-year old woman who developed postpartum a liver disease and HELLP syndrome with fatal complications. She carried the homozygous p.(Gln358Lys) variant located in exon 11 [18]. The authors considered that the combination of the metabolic stress of pregnancy and the genetic predisposition overloaded enzyme capacity and caused the accumulation of potentially toxic fatty acid metabolites in the maternal circulation with subsequent damage to the maternal liver. Variants p.(Val282Asp) (at the homozygous state), and p.[(Ile305Asn;Arg291*)] (compound heterozygous) have been described in pure neuromuscular phenotype with late onset (12–13 years old) and milder course [19]. However, neither retinopathy nor cardiomyopathy were reported in any of these patients. Finally, other variants in the large French cohort described by Boutron and colleagues were identified in patients with late-onset neuromyopathic forms [1]: p.(Ile305Asn) and p.(Arg235Trp) located respectively in exon 8 and 9. All these uncommon late-onset phenotypes are associated with variations located in a region ranging from exons 8 to 11 corresponding to the linker domain connecting the NH_2_-terminal hydratase domain (exons 3 to 7) to the C-terminal 3-hydroxyacyl-CoA dehydrogenase domain that encompasses the NAD binding domain (exons 12 to 20) [20]. In our case, the p.(Glu510Gln) was associated with another missense variation, never reported before, the p.(Gly370Arg) located at the beginning of the NAD binding domain (exon 12). This novel variant is located in close proximity to the linker domain, and the substitution of glycine to arginine, which is a larger and positively charged amino acid, should clutter and cause disorganization of the 3D structure of the protein (Fig. 3). Ibdah and coworkers showed that missense variants in exon 9 of the alpha-subunit altered the expression of both alpha and beta subunits, and therefore the MTP complex formation which is essential for the stabilization of both units [21]. *HADHA* missense variants in the region between the hydratase and the NAD binding domain (the linker domain) are probably responsible for degradation of alpha and beta subunits, and thus for reduced enzyme capacity to bind or to transfer long-chain fatty acid substrates. Consequently, these latter accumulate, become toxic for cellular structures and induce multiple organ dysfunction. However, MTP activity allows sufficient expression of the protein for carriers to reach old age without clinical manifestations. We could speculate that the 5′ end of the NAD binding domain, where is located the p.(Gly370Arg) variant, might not be functionally as important as the neighboring domains. The bi-allelic combination of the two variations found in our patient, differing each in localization and pathogenicity, could explain late-onset, initial slow progression and the sudden cardiac dysfunction.

The last explanation for a slow evolution could be a protective effect of genetic or environmental modifiers on the deficient metabolic pathway. Indeed, a regular food intake with no fasting habits promote the glycolytic pathway and avoid the use of mitochondrial beta-oxidation and therefore overload. One might assume that our patient, during his entire childhood and adolescence, had a “protective” lifestyle that may have slowed down the accumulation of cardiotoxic long-chain 3–hydroxyAcyl-CoA before reaching a threshold for decompensation. The macular manifestations may have occurred earlier than the other features because fatty acid oxidation is a main energy provider in the retina [8], [9]*.*

Our patient did not benefit from newborn screening (NBS) since NBS in France still does not include screening for LCHAD. Most countries performing LCHAD screening use 3-hydroxypalmitoylcarnitine (C16-OH) as primary marker. Medical literature reports different C16-OH thresholds ranging from 0.03 to 0.18 μM, according to countries [22]. Could our patient have been detected by NBS? In the absence of neonatal C16-OH determination, we confronted the various reported NBS cut-offs to the C16-OH value (0.13 μM) at diagnosis in our patient, as it reflects the metabolic status in absence of any specific treatment or diet. Considering this value, we hypothesize that the patient may not have been detected by NBS depending on thresholds used in different countries. Because LCHAD is a rare disorder, this highlights the importance of a low threshold to detect mild or atypical forms.

Triheptanoin aimed to provide anaplerotic metabolites, to replace deficient TCA cycle intermediates and improve energy metabolism. Several studies have shown the benefit of triheptanoin treatment in patients with cardiomyopathy-associated with long chain-fatty acid oxidation disorders, mainly in children, and in association with cardiovascular treatment [23]. It is difficult in our patient to distinguish between the effectiveness of enhanced cardiovascular therapy and specific metabolic treatments. However, it is unusual that intensification of cardiovascular drug regimen may improve heart failure, initially unresponsive to treatment, to the extent that heart transplantation was no longer deemed necessary. Therefore, we believe that specific metabolic management, in association with an optimized cardiovascular drug regimen played a role in favorable outcome.

## 5 Conclusion

This case illustrates the difficulties in making clinical and molecular diagnosis at the early stages of a disease with mild and/or unusual features. It also highlights the necessity of close interaction between clinical specialists in order to provide a full clinical picture which can comprise elements evocative of metabolic disease phenotypes leading to more efficient and targeted biochemical and molecular diagnosis. This is particularly true concerning atypical presentations such as ours given that we can expect a large number of underdiagnosed mild/late-onset phenotypes, because of a very high prevalence of the particular *HADHA* p.(Glu510Gln) allele in the population of Northern Europe [24]. Indeed, our patient illustrates that in the presence of unexplained macular dystrophy and/or unexplained hypertrophic or dilated cardiomyopathy, the carnitine status and the acylcarnitines profile should be determined to exclude or confirm a beta-oxidation disorder. Finally, in case of positive diagnosis, specific metabolic treatment with triheptanoin in combination with cardiovascular therapy should be considered.

## Fundings

This research did not receive any specific grant from funding agencies in the public, commercial, or not-for-profit sectors.

## Institutional review board statement

The study was conducted according to the guidelines of the Declaration of Helsinki and in accordance with the French bioethics law.

## Informed consent statement

Informed consent and oral agreement for publication was obtained from the subject involved in the study.

## Data availability statement

All data are contained within the article or supplementary material.

## CRediT authorship contribution statement

**Anne-Frédérique Dessein:** Conceptualization, Investigation, Data curation, Writing – original draft, Writing – review & editing. **Eléonore Hebbar:** Investigation, Writing – review & editing. **Joseph Vamecq:** Investigation, Data curation, Writing – review & editing. **Elodie Lebredonchel:** Investigation, Writing – review & editing. **Aurore Devos:** Investigation, Writing – review & editing. **Jamal Ghoumid:** Investigation, Writing – review & editing. **Karine Mention:** Investigation, Writing – review & editing. **Dries Dobbelaere:** Investigation, Writing – review & editing. **Marie Joncquel Chevalier-Curt:** Investigation, Writing – review & editing. **Monique Fontaine:** Investigation, Writing – review & editing. **Sabine Defoort:** Investigation, Writing – review & editing. **Vassily Smirnov:** Investigation, Writing – review & editing. **Claire Douillard:** Investigation, Writing – review & editing. **Claire-Marie Dhaenens:** Conceptualization, Investigation, Data curation, Writing – original draft, Writing – review & editing, Supervision.

## Declaration of Competing Interest

No conflicting relationship exists for any author.
